# Control of Cracking in Textile Reinforced Concrete with Unresin Carbon Fibers

**DOI:** 10.3390/ma13143209

**Published:** 2020-07-18

**Authors:** Rui Neves, Diogo Felicíssimo

**Affiliations:** Barreiro Technology School, Polytechnic Institute of Setúbal, Barreiro, R. Américo Silva Marinho, 2839-001 Lavradio, Portugal; diogo.felicissimo@estudantes.ips.pt

**Keywords:** TRC, crack control, tensile testing, carbon fiber, concrete, sustainability

## Abstract

Textile reinforced concrete (TRC) is an emerging construction material with interesting potential concerning sustainability, providing corrosion-free and lightweight solutions. Ordinarily, fiber bundles, impregnated with resin, are used. In this research the performance of reinforcement with unresin fibers is investigated. Control of cracking is considered the key performance factor and is assessed through tensile testing. However, economic and environmental aspects are addressed as well. Then, four different mixes/matrices were considered, without the addition of special/expensive admixtures. TRC ties were subject to direct tension tests, with load and deformation monitoring to assess the influence of mechanical reinforcement ratio on the cracking, failure and toughness of these composites, as well as of the matrix properties on the maximum load. It was observed that at a macro-level TRC behaves like conventional reinforced concrete, concerning crack control. Based on the maximum loads attained at the different composites, it was found that this particular TRC is economically viable. It is suggested that matrix workability may influence the maximum load.

## 1. Introduction

### 1.1. Background

Reinforced concrete structures have a major role in the construction of buildings and infrastructures. Ordinary concrete is a very versatile material, adaptable to complex geometries, economic and manufacturable on site. However, it has a brittle behavior and limited tensile strength. To overcome these drawbacks, it is often reinforced with steel, which is ductile and has good tensile strength. In conventional reinforced concrete, steel bars are embedded in concrete in strategic positions, to optimize its reinforcing effect. This solution makes the production process more complex, as it is necessary to properly place the reinforcement and brings a significant problem: steel corrosion, which impairs the durability of conventional reinforced concrete elements. To mitigate these problems, several alternatives have been tried.

To diminish the complexity of the production process, long/continuous reinforcing bars may be replaced by short/discontinuous fibers, added to the concrete at the mixing stage, together with the other concrete constituents. This solution is called fiber reinforced concrete. Although it eases the production of concrete and provides some tensile strength, the reinforcement efficiency is limited, as fibers are randomly dispersed in the concrete, while the acting stress fields generally have preferential directions. Thus, to have an equal resisting bending moment in a conventional reinforced concrete beam and in a fiber reinforced concrete beam, a higher reinforcing ratio is required in the latter. Furthermore, the mixing and placing of fiber reinforced concrete with fiber volumes required to achieve current resistant bending moments is impractical. Only special production techniques, like SIFCON [1], allow structural fiber reinforced concrete elements to be produced. Another handicap of fiber reinforced concrete is that due to the short length of the reinforcements, it is often not possible to mobilize their full strength, because they debond and slip before they yield or break.

To avoid corrosion problems, stainless steel or epoxy-coated steel bars or fiber reinforced polymer (FRP) rods have been used instead of carbon steel rebars [2,3]. The two first alternatives shorten deterioration by corrosion but do not fully eliminate corrosion problems once iron is still present. The latter option is corrosion-free and provides higher strength-to-weight ratios. Nevertheless, under high temperatures, FRP properties are quite impaired [4] and FRP cannot be bent in the field (the same is for epoxy-coated steel bars) [5]. Finally, all these alternatives have a high initial cost [6].

### 1.2. Textile Reinforced Concrete

Textile reinforced concrete (TRC) is a composite material that consists of conveniently aligned continuous high strength fibers and fine-grained concrete (matrix). Even though the most common designation is TRC, the usual maximum aggregate grading ranges between 1 and 2 mm [7,8]. Then, it is actually textile reinforced mortar (TRM), a designation that is also found in the literature. As fibers are usually arranged as woven or knitted fabrics, designations like fabric reinforced cement composites (FRCC) and fabric reinforced cementitious matrix (FRCM) can also be found.

Research on the reinforcement of cement-based matrices with long/continuous fibers started on the 1960s [9]. Scheerer et al. [10] mention that the first patents for this new building material were issued in the 1980s. At the beginning of the 21^st^ century, two research centers were established in Germany, with the purpose of carrying out research and development on TRC [11].

Within the major features of TRC are the absence of corrosion problems and the ability to adapt to complex shapes, due to reinforcement flexibility [12]. The fact of being corrosion-free allows volume cutbacks, as the reinforcement cover is ruled by bond requirements instead of requirements for the protection of reinforcement against the action of corrosive agents. The spared volume is considerable in elements where the cross-section thickness is constrained just by cover requirements, as is the case for lightly-loaded and non-load-bearing elements. Savings up to 75% have been claimed by Curbach et al. [13]. Laiblová et al. [14], in a comparison between TRC and conventional reinforced concrete, through Life Cycle Assessment, have considered a volume reduction of 70%. They defend the idea that TRC has potential for use in sustainable construction, as well as the claim that, besides reducing production costs, TRC also brings savings in transportation costs as the elements are lighter.

TRC has been applied in the production of façades, roofing and wall panels [11,15,16], parapets [17], balcony slabs [10] and permanent formworks [12]. Still within the frame of non-load-bearing and lightly-loaded elements, it is also believed that TRC is suitable for the production of fencing elements. Bold applications of TRC also exist: the Döllnitz Creek Bridge [18], the Lautlingen Bridge [19], the Bridge over the Rotach River [20] and a set of four roof shells with 3.5 m cantilever spans at the campus of RWTH Aachen University [21]. The most popular application of TRC is the retrofitting of existing structural elements [22,23,24], which encompasses slightly different concepts from the previous examples and therefore is considered outside of the scope of the present research.

### 1.3. Aims and Scope

Commonly, reinforcement in TRC is provided by prepreg fibers. This means that the fibers are pre-impregnated with a resin. With this, a group effect is generated within a bundle of filaments and higher strengths are attained. The strength of an unresin bundle ranges between 10 and 25% of a single filament strength [25,26]. This happens because there is no uniform state of stress within the bundle. Some fibers are slack while others may be already tensioned [25,27,28]. Further, some filaments may be misaligned, and others twisted [27]. Therefore, it is not possible that the response of a bundle with *n* filaments is *n* times the response of a filament. If the bundle is impregnated with resin, its strength varies from 35 to 60% of the strength of a single filament [7], depending on the weight content of resin in the prepreg [29]. Besides the strength increase, in relation to unresin fibers, fiber impregnation makes the reinforcement placing (in regular elements, e.g., walls) easier, due to the increase in transverse stiffness, and it is also claimed to improve the bond with the cementitious matrix [24,30,31].

However, unresin carbon fibers have some advantages over impregnated fibers, such as being less costly, lighter, less susceptible to high temperatures and easier to adapt to complex geometries. Moreover, the use of resins raises eco- and human toxicity concerns. Studies have proven that Bisphenol A, used in the production of resins, even in low doses, causes alteration in brain chemistry and the immune system, among others, in several animal species [32].

At the beginning of the 2000s, there was some investigation of concrete reinforcement with unresin continuous carbon fibers [25,28,33,34,35], which has since been discontinued. One may speculate that this was due to the results not being very promising. Still speculating, such discouraging results may be linked to the fact that the research was oriented to the application of unresin carbon fibers in heavy-loaded elements. At this point, it shall be emphasized that the existing TRC bridges are footbridges.

One key for sustainability is the optimization of civil engineering materials. This optimization must be fostered through application and design.

Concerning application, different materials can be considered for one construction. The advantages and disadvantages of each solution will be weighed, to ensure the most appropriate choice. Lightly-loaded or non-load-bearing elements, where element thickness is ruled by minimum reinforcement cover requirements (if reinforcement is provided by steel bars), is where the replacement of conventional reinforced concrete by TRC brings more benefits. Further, these elements are generally precast. One of limitations of TRC is that its production on site is not recommended [36]. Therefore, this type of application is the optimal cluster for TRC.

In the context of fiber reinforced concrete design, a common parameter is the critical fiber volume. This parameter, the amount of fiber per unit volume required to resist the cracking load, is also considered for TRC. According to Hartig et al. [37], if the fiber volume is 30% more than the critical, it is ensured that the tensile stress-strain curve is trilinear. This type of load-bearing behavior is the prevailing one among the results found in the literature. Thus, this means that the tested TRC has, at least, 30% more reinforcement volume than the minimum required for control of cracking. The ruling criterion for the design of lightly-loaded or non-load-bearing elements is that the existence of a minimum reinforcement ratio is required, just to ensure the physical integrity and the functionality of the element, as well as the avoidance of unsightly appearance, through control of cracking. If TRC is to be used in such elements, then its minimum reinforcement ratio should be investigated.

Still fostering sustainability, the current design will also seek materials inherently nontoxic to biological systems and for the reduction of waste.

Therefore, the aim of this investigation is to resume the research on TRC with unresin fibers, instead of prepreg fibers, which are hazardous to health and more difficult to recycle. Contrary to what has been found in the literature, the main focus is set on the minimum reinforcement ratio for control of cracking, as it is the ruling design criterion for the excellence cluster of TRC applications. Still aiming at the optimization of benefit vs. cost, and also in opposition to what has been found in the literature, the addition of special admixtures to the matrix and even microfibers, which have been proven to enhance TRC performance [8,21,38,39,40,41] but also contribute to increasing the initial cost per unit volume of TRC, will be avoided in this research.

Developing suitable TRC with unresin fibers is expected to bring economic, environmental and health benefits. This suitability will be evaluated through the tensile behavior of the tested composites. An original system to transfer the load from the testing machine to the specimens is essayed.

Although it is claimed that TRC may be more economical than conventional reinforced concrete, a literature review did not reveal any explicit cost analysis. Therefore, the experimental assessment of the tensile behavior is followed by an analysis of economic and environmental costs.

## 2. Materials and Methods

Hereunder, the most relevant characteristics of the materials used in this research are presented. Information is also provided about specimen manufacturing and testing.

### 2.1. Materials

#### 2.1.1. Matrix (Concrete/Mortar)

**Figure 1:**
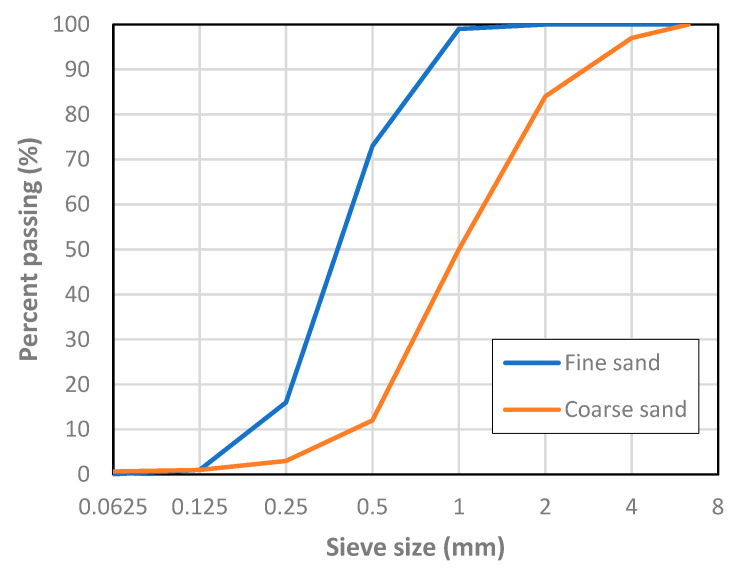
Aggregate particle size distribution.

Cementitious matrices composed of cement, sand, water and superplasticizer were designed. The cement type was CEM I, ordinary portland cement, of the strength class 42.5R, conforming to EN 197-1 [42], with a specific gravity of 3.14 g/cm^3^. Fine and coarse natural siliceous sands with a specific gravity of 2.63 g/cm^3^ were used. Figure 1 depicts the particle size distribution of both sands. To prevent scale effects, the maximum aggregate size, 4 mm, was less than 1/3 of the least dimension of the specimens. The larger than usual grain size also allows matrix shrinkage to be limited [15].

The criterion to define the different mixes was to obtain an increasing matrix strength (from A60 to A35). In this way, decreasing mechanical reinforcement ratios were achieved, keeping the tensile specimen dimensions constant and the amount of reinforcement as well (see Section 2.2). The proportions of the mix constituents are reported in Table 1. A superplasticizer was used in the two mixes with the lower water-cement ratios.

#### 2.1.2. Reinforcement (Carbon Fiber)

To reinforce the matrix, carbon fibers (Fisipe, Lavradio, Portugal) were used. These fibers were produced from oxidation and carbonization of polyacrylonitrile (PVA) fibers, supplied in a bundle with linear mass of 3300 tex, containing 50k filaments with a 7 μm diameter. According to the producer information, these fibers have a specific gravity of 1.81 g/cm^3^, elasticity modulus of 240 GPa and tensile strength and elongation at break of 4 GPa and 1.7%, respectively.

The received fiber was subject to direct tension tests, carried out on bundle sections. To prevent shear rupture of the fiber, steel wool bits were placed between the bundle and grips [43]. A displacement ratio of 0.5 mm/min was applied [44]. From the tests, a characteristic value of 1.94 kN for ultimate load was obtained. An average stress may be calculated dividing this load by the area of the bundle cross-section (A_f_). Following Colombo et al. [45], that area can be obtained from the bundle linear mass (m_l_) and fiber specific gravity (γ_f_) through

(1) $$A_{f}\left[{\mathrm{mm}}^{2}\right]=\frac{m_{l}\;\left[tex\right]}{1000\times \gamma_{f}\;\left[\frac{g}{{\mathrm{cm}}^{3}}\right]}$$

From this, a value of 1.83 mm^2^ for A_f_ is found, and a corresponding tensile stress of 1.06 GPa is computed. Therefore, the bundle efficiency is 0.25.

### 2.2. Preparation of Specimens

**Figure 2:**
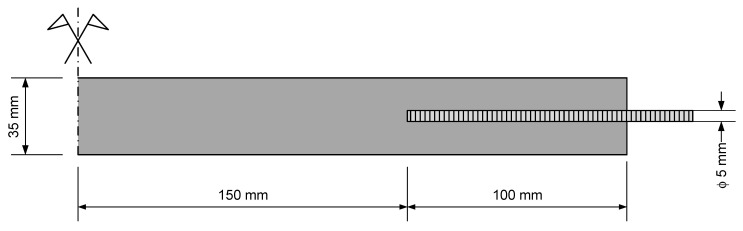
Sketch of the ties.

For each mix, three prisms of 40 × 40 × 160 mm^3^ were cast in steel molds, compacted by means of a vibrating table. After demolding (at 24 h) these specimens were kept in water at 20 °C. Further, four 15 × 35 × 500 mm^3^ ties were cast for uniaxial tensile testing. The dimensions of the ties are within the range found in the literature [37,45,46,47,48,49,50,51]. The thickness is more than three times the maximum aggregate size, to avoid scale effects. The width-to-length ratio of the ties ensures the development of stabilized cracking, if the reinforcement ratio accomplishes the minimum required for control of cracking and makes the Poisson effect quite limited [47]. Based on the approach followed by Jaccoud [52], at both tops threaded rods with 5 mm diameter were embedded in the specimen to a length of 100 mm (Figure 2). The rod diameter and embedded length were designed to ensure a proper load transfer from the universal machine clamps to the specimens.

Three of the four ties were reinforced with unresin carbon fiber, at a fiber content of 0.35% (vol./vol.), provided by a single bundle, from top to top, thus overlapping with the rods. The required anchorage length of the fiber bundle was assessed through preliminary pull-out tests. The manufacturing process was the hand-lay-up or laminating [40,41,45], i.e., the matrix was poured into the molds up to the intended position of the reinforcement layer, then the reinforcement was placed, and afterwards another layer of matrix was poured until the matrix started to flow out of the molds. After pouring each matrix layer, the specimens were compacted using a vibrating table. Finally, the casting surface was finished using troweling, eliminating the surplus of matrix all over, so that the thickness of the ties is homogeneous.

Due to the slenderness of the ties, to avoid accidental microcracking at formwork removal, these specimens were demolded only 48 hours after casting. Then, the ties were kept in water at 20 °C.

### 2.3. Test Methods

In the following, the procedures to characterize matrix workability and strength and to assess tensile behavior of TRC are described.

#### 2.3.1. Workability

The workability of the fresh mixes was measured according to ASTM C1437-13 [53]. The spread of a truncated cone of mortar in a flow table subject to jolting was measured in two orthogonal directions. Due to mortar fluidity, the number of strokes was limited to ten, so that the spread did not overflow the table.

#### 2.3.2. Matrix Strength

The compressive, flexural and tensile strength of the cementitious mixes was evaluated.

Flexural strength was assessed through testing of three prisms of 40 × 40 × 160 mm^3^ per mix at an age of 28 days, following EN 196-1 [54]. The specimens were subject to three-point bending, applying a load rate of 50 N/s at mid-span. Afterwards, the resulting six half-prisms were subject to compression tests, at a load rate of 2400 N/s, following the same standard. Both tests were run on a Matest cement compression flexural testing machine (Matest, Treviolo, Italy), equipped with a load cell with a maximum capacity of 250 kN for compression testing and with a load cell with a maximum capacity of 15 kN for flexure testing.

The tensile strength was evaluated though a direct tension test on unreinforced ties, whose details are described next (Section 2.3.3).

#### 2.3.3. Direct Tension

**Figure 3:**
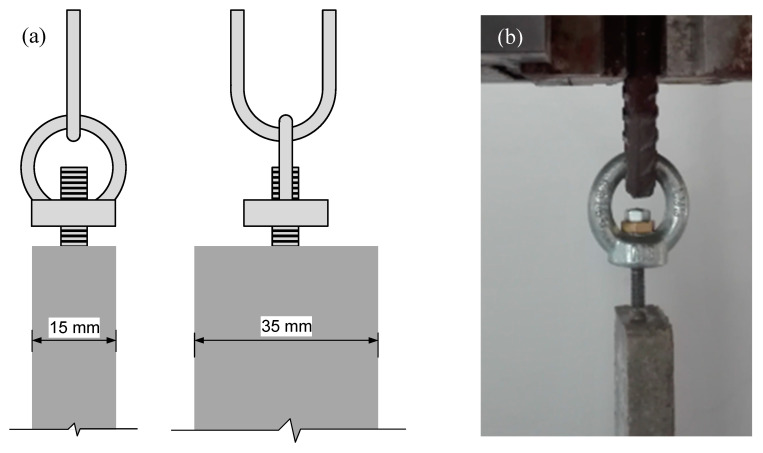
Sketch (**a**) and view (**b**) of the specimen-clamps connection.

Direct tensile testing is arduous and therefore not very common [55,56,57]. Although there are reference guidelines for tensile testing of TRC [58], there is no standard. There are several techniques to apply the tension load to the specimen and to prevent secondary effects like bending and shear [30,58,59]. In the present research, 3D hinges were introduced between the threaded rod and the testing machine clamps. These 3D hinges were materialized by means of assembling two bidimensional hinges in orthogonal planes (Figure 3).

**Figure 4:**
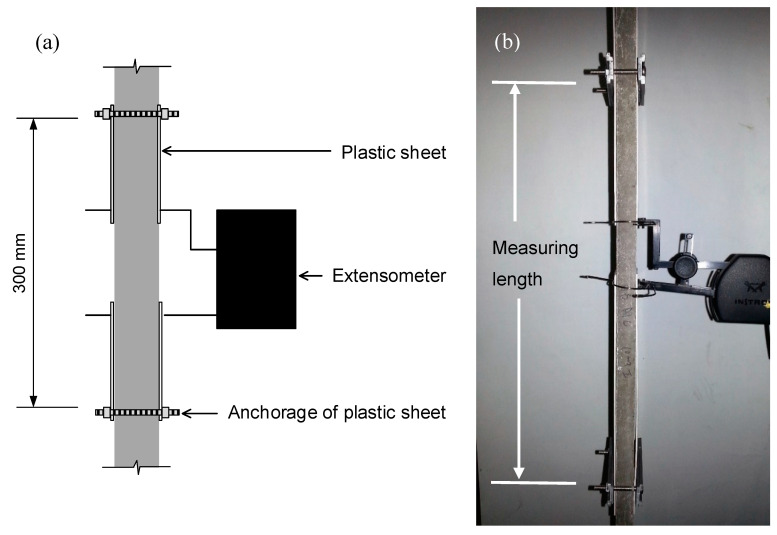
Sketch (**a**) and view (**b**) of the deformation measurement setup.

Tests were conducted using a screw-driven universal test frame Instron 5900-R (Instron, Norwood, MA, USA), equipped with a 10 kN load cell capacity (class 0.5). The tension load was applied under displacement control at a rate of 0.5 mm/min, common within this type of testing [33,40,50]. To improve the accuracy of the axial deformation measurement, a clip-on extensometer Instron 2630-112 (Instron, Norwood, MA, USA) with a gauge length of 50 mm and a travel range between −2.5 and +25 mm was used. The accuracy (0.5%) is relative to the gauge length. Thus, an accuracy of ±25 µm is ensured. The measuring length was 300 mm, to encompass any crack formed during the test. Given the difference between the gauge length and the measuring length, the extensometer was clipped over plastic sheets, which were anchored to the specimen at the limits of the measuring length, as depicted in Figure 4.

All direct tension tests were carried out when the specimens were 28 days old.

## 3. Results

In this section, the results obtained in the different experiments are reported. Those related with the matrix properties are briefly discussed here, while tensile behavior is the object of a detailed discussion in 4.1.

### 3.1. Matrix Properties

**Figure 5:**
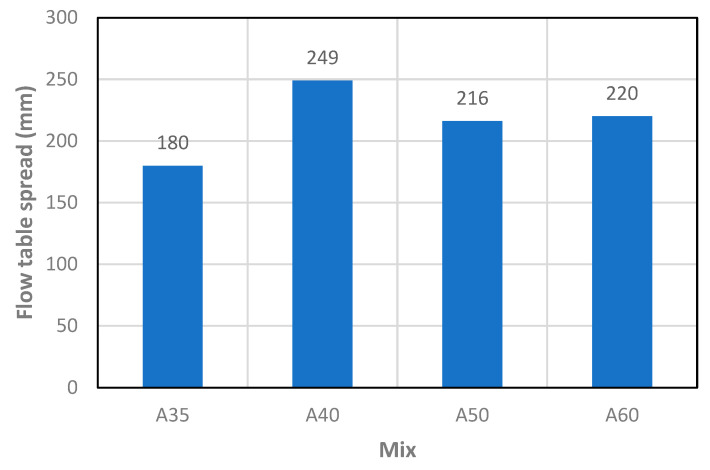
Matrix workability obtained from flow table test.

Figure 5 shows the average spread diameter of each mix. Considering two groups of mixes, one for mixes with superplasticizer (A35, A40) and the other from mixes without superplasticizer (A50, A60), in each group the workability increases with the water-cement ratio as expected.

**Figure 6:**
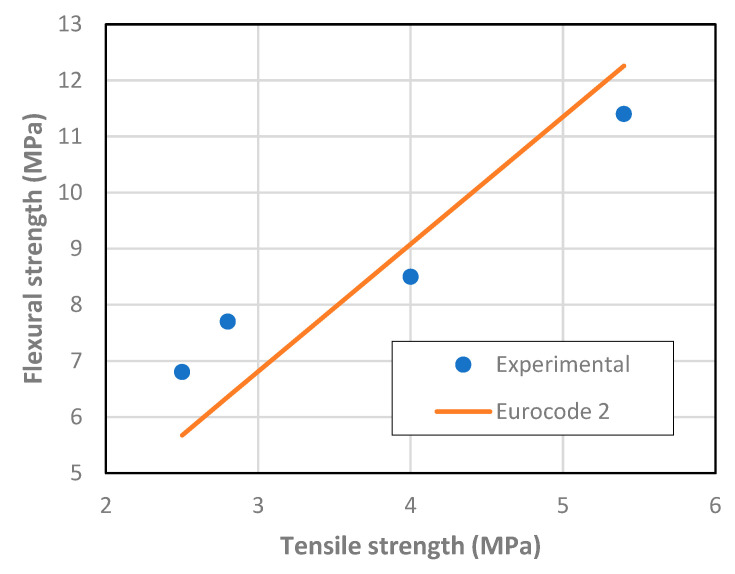
Relationship between matrix tensile and flexural strength.

**Figure 7:**
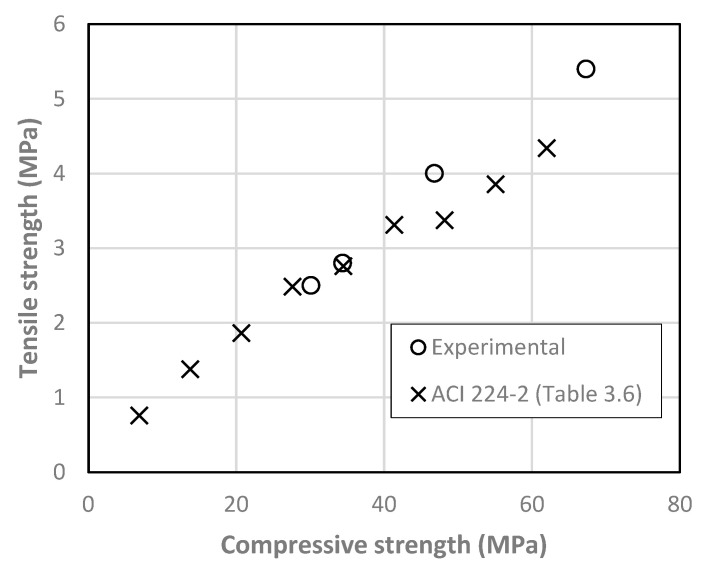
Relationship between matrix compressive and tensile strength.

The strength results are summarized in Table 2. There are consistent increases in strength with the reduction of the water-cement ratio. Further, in Figure 6 and Figure 7, the relationship between the tensile strength and the flexural and compressive strengths are shown, respectively.

A good agreement is found with existing relationships in reference documents [60,61]. From Figure 7 it can be roughly estimated that tensile strength corresponds to 8% of the compressive strength.

### 3.2. Tensile Behavior of TRC

**Figure 8:**
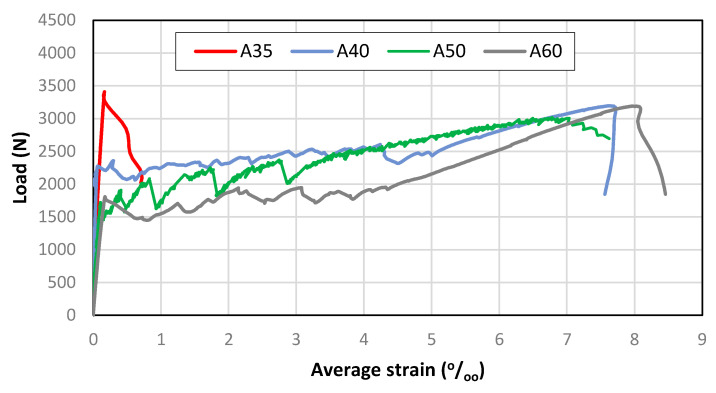
Load-deformation curves of TRC ties.

**Figure 9:**
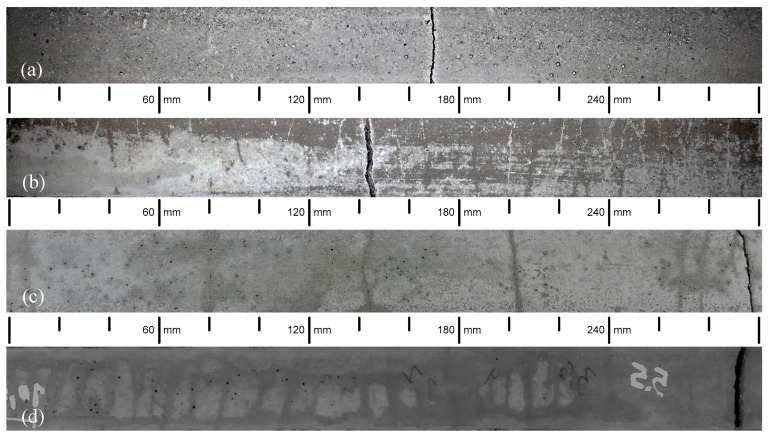
View of the ties after tensile testing: (**a**) A35, (**b**) A50, (**c**) A40, (**d**) A60.

According to De Santis et al. [36], the most important parameters from TRC tensile testing are the stress at first crack (σ_cr_), the crack spacing (S_rm_), the ultimate load (F_u_) and the corresponding deformation (ε_u_). These values are presented in Table 3 and are taken from the load-deformation curves (Figure 8) and from the cracking pattern of the ties after testing (Figure 9). It must be mentioned that in one or two ties from each mix, crack localization occurred, near the limit of the measuring length. This type of failure also occurred in the experimental investigation of Carozzi et al. [62] and here is attributed to a difference between the estimated and the required anchorage length. As reported in 2.2, the design of the anchorage length was based on pull-out tests. However, the thickness of the pull-out specimens was higher than the thickness of the ties. It is judged that the higher thickness of the former caused some confinement at the developing microcracks on the reinforcement-matrix interface. That did not happen on the thinner ties, allowing the microcracks to develop more easily and limiting the bond strength between the reinforcement and the matrix. Nevertheless, the ties where there was no crack localization (before saturation crack spacing) had quite similar behavior if composed of the same matrix. Figure 8 depicts data from one tie for each matrix and Figure 9 the view of that tie after testing.

The energy absorption capacity and ductility are also important in several TRC applications. Therefore, strain energy and ductility are evaluated. Strain energy (U) is computed as the integral of the load-elongation product until 70% of the ultimate load at the softening branch. Ductility is quantified through the ratio between the average strain at the ultimate load and the strain at cracking. The results of these two parameters are presented in Table 4.

## 4. Discussion

### 4.1. Tensile Behavior

The cracking load of the different TRC varied generally according to the matrix strength. As expected, this parameter was controlled by the lowest ultimate strain of the composite constituents, which was the matrices’ ultimate strain. However, the lowest cracking load was observed in A50. This is attributed to a presumable defect in the tie, as the response after cracking was hardening and the second crack appeared at a load already superior to that of A60 cracking load.

Concerning the ultimate load, the highest value was for A35, while A40 and A60 composites had quite similar ultimate loads. The ultimate load of A50 was a bit lower but still comparable with those of A40 and A60. The difference between these two groups lies in the controlling factor of the ultimate load. For A35 it was matrix strength, whereas for the others it was reinforcement load-bearing capacity. In A40, A50 and A60, after cracking, the fibers were able to withstand the imposed strain without a significant loss of load-bearing capacity, transferring stress to the matrix that caused further cracking. In contrast, in A35, the cracking load was too high to be sustained by the fiber bundle alone at the cracked section, causing the composite to fail without further cracking. Furthermore, in A35 a single crack was formed, while in the others there were multiple cracks.

Observing the ultimate loads of A40, A50 and A60 composites, it is estimated that the fiber bundle can sustain a load of around 3 kN when embedded in cementitious matrices. The ultimate load of A50 being lower than those of A40 and A60 is attributed to a poorer fiber-matrix bond. It is judged that the inferior bonding is caused by a lower fluidity of the matrix that has less penetrated the fiber bundle. The mix A50 was the one with the lowest water-cement ratio of those without superplasticizer. Nevertheless, a similar value is found for the fiber bundle in A35. At cracking of A35, the load transferred to the bundle (3.4 kN) was higher than its load-bearing capacity, which led to the immediate failure, without further cracking.

In A40, A50 and A60, cracks were formed successively until crack saturation was attained. Afterwards, there was strain hardening and the deformation became concentrated in one of the already existing cracks, as can be observed in Figure 9, where there is a wider crack in every specimen. This crack localization is common in TRC [33,62] and may be attributed to the unevenness of reinforcement efficiency at the different cracks, causing a larger deformation in the crack where the stiffness/reinforcement efficiency is lower. These three composites exhibited a load-deformation behavior that fits high modulus/medium bond reinforcement TRC [7]. Still within the frame of cracking pattern, it is noticed that crack development comprised regular cracks and microcracks, some of them could only be visually detected for a short timeframe during the drying phase after wetting the specimens. This microcracking is usual in TRC subjected to tension and corresponds to the minor load drops in multiple crack formation phase present in Figure 8. The bundle is not fully penetrated by the fresh matrix due to filtering effects caused by the narrow spacing between filaments [37], and therefore, some filaments are not in contact with the matrix throughout and are poorly bonded to the matrix at some parts. Furthermore, the number of filaments poorly or not bonded to the matrix may also vary from section to section. The degree of bonding is not therefore homogeneous along the ties’ length. Thus, it will be natural for cracks to form with different widths. The strain hardening increased with the mechanic reinforcement ratio, as suggested in [37].

Similar crack spacing was observed in A60 and A40, while A50 showed a smaller number of cracks, thus a wider spacing. This is attributed to the already discussed weaker fiber-matrix bond, which requires longer lengths to transfer the load carried by the fiber at cracks to the matrix. Crack spacings in TRC like those obtained in this research have already been reported [37,49,63].

Regarding average strain at peak load, similar values are present in A60 and A40, while for A50 the value is a bit smaller (in agreement with the ultimate load variation). Obviously, the strain at peak load for A35 is a lot smaller because it corresponds to matrix strain at maximum tensile stress. 

Therefore, there is a significant difference between the tensile behavior of A35 and the other tested solutions, regarding ductility. This is evidenced by the large differences in strain energy and in the ratio considered to quantify ductility between A35 and the other TRC composites.

All TRC composites failed by fiber pull-out. After the peak-load there was a stiffness drop that triggered automatic stop of the test. Although the crack localization caused local strains that were higher than the fiber ultimate strain, the ties were still not in separated pieces. This happens because when the reinforcement is provided by fiber bundles, the state of strain is not the same at all filaments. There is a strain lag [64] and further a telescopic pull-out behavior [45]. The outer filaments are more in contact with the matrix than the inner ones. The latter tend to slip, transferring strength only by friction. This makes it possible that a composite constituted by two fragile materials can present a ductile behavior. Furthermore, it is noticed that the load-bearing capacity of the considered fiber bundle is increased by around 50% when embedded in the matrix as compared with the bare bundle. This is attributed to the fact that the strain lag is less when the fibers are embedded in other material, thus resulting in more filaments resisting the load simultaneously.

Comparing the tensile behavior of TRC with that of conventional reinforced concrete, there are similarities and differences. In both, there is a mechanic reinforcement ratio threshold that enables control of cracking. The amount of reinforcement should be capable of withstanding the energy that is released when the matrix cracks. In this research, the mechanical reinforcement ratio of A35 is under that threshold, while that for A40, A50 and A60 is above.

The smaller reinforcement diameter of TRC, accompanied by a group effect (narrower reinforcement spacing), does not allow the application of the existing analytical models for the simulation of crack behavior of conventional reinforced concrete to TRC. Furthermore, the group effect also causes heterogeneity concerning the fiber involvement by the matrix. This is a complex issue, as the fresh state matrix properties also play an important role in the ease with which it can surround fibers. Moreover, the number of filaments in a bundle also influences the ease by which the matrix penetrates the bundle [65]. Nevertheless, the decrease of crack spacing with the reinforcement (fiber in TRC) diameter is still valid [60]. Likewise, the condition of avoiding brittle behavior at cracking, through ensuring a minimum amount of reinforcement for which load at first cracking is not higher than the reinforcement ultimate load, is applicable to TRC and will be used next.

### 4.2. Economic and CO_2_ Emission Analysis

The economic analysis will not be linked to any particular currency, as it will be based on relative costs. Let us consider steel as the reference material concerning cost. Following Meredith et al. [66], the ratio between carbon fiber and steel cost (wt./wt.) is 22. According to Ohta et al. [25], the cost of impregnated fiber is five times higher than the cost of unresin fibers. Based on the cost data from Alreshaid et al. [67], the cost ratio between concrete and steel is 0.09 (wt./wt.). Once structural design is based on geometric quantities, the cost ratios of carbon fiber-steel and concrete-steel will be converted to (vol./vol.), considering densities of 1.81, 2.32 and 7.85 for carbon fiber, concrete and steel, respectively. The information is summarized in Table 5.

For the mechanical properties, it is assumed that concrete has a tensile strength f_ct_ = 3 MPa and that the yield stress of steel is 500 MPa. Based on the present experimental work, it will be fair to assume an ultimate load F_u_ = 3 kN for a bundle of unresin carbon fibers totaling 1.83 mm^2^ of fiber cross-section embedded in the cementitious matrix. Based on the results from Donnini et al. [30], a ratio of 1.7 between the tensile strength of embedded unresin and impregnated carbon fibers is roughly estimated.

The structural element is assumed to be lightly-loaded and its thickness is ruled by durability constraints, for conventional reinforced concrete, and ruled by bond requirements in TRC. Then, as in [14], thicknesses of 60 mm and 18 mm are considered for the conventional reinforced concrete and for the TRC solutions, respectively. For the sake of simplicity, the remaining dimensions of the element (width and length) are 1 m.

To ensure control of cracking, the reinforcement must be capable of bearing the force that is transferred when a crack opens in the matrix. Therefore, for the conventional reinforced concrete, a concrete cross-section, A_c,CRC_, requires a steel area A_s_ = (3/500) × A_c,CRF_. As for this solution, A_c_,_CRC_ = 0.06 m^2^, then A_s_ = 35 × 10^−5^ m^2^.

Following the same principle for TRC, a concrete cross-section, A_c,TRC_, requires a number of unresin carbon fiber bundles N_UCF_ = (f_ct_ × A_c,TRC_)/F_u_ = (3 × 10^3^ × A_c,TRC_)/3. As for this solution A_c_,_TRC_ = 0.018 m^2^, then N_UCF_ = 18, which corresponds to an area of 32.94 mm^2^. Considering the previously estimated efficiency of impregnated carbon fiber, still for the TRC solution the required reinforcement area with impregnated fibers is calculated through A_ICF_ = A_UCF_/1.7, which returns A_ICF_ = 19.38 mm^2^. The quantities and solution costs of the materials are presented in Table 6.

The results in Table 6 indicate that TRC with unresin carbon fibers is the most competitive solution in terms of cost, with savings of 70% in relation to conventional reinforced concrete and of 7% when compared to TRC with impregnated carbon fibers.

Regarding CO_2_ emissions, the ratios presented in Table 7 are based on information collated from the literature [68,69]. Then, the quantities in Table 6 are converted from m^3^ to kg, using the previous densities. Within this frame, a 45% content (wt./wt.) of resin in the impregnated carbon fibers is assumed. Finally, total CO_2_ emissions for each solution are computed and presented in Table 8.

TRC with unresin fibers reduces CO_2_ emissions by 70% when compared with conventional reinforced concrete. However, the solution with the lowest CO_2_ emissions is TRC with impregnated fibers, which has 10% less emissions than TRC with unresin fibers. Nevertheless, the use of resin encompasses additional environmental issues, as stated in 1.3, whose quantification falls outside of the scope of the present research. 

## 5. Conclusions

A brief retrospective of concrete reinforcement was provided. Textile reinforced concrete was identified as a solution that meets sustainability criteria. Precast lightly-loaded or non-load-bearing elements, whose thickness, in the case when they are made of conventional reinforced concrete, is ruled by durability concerns, have been identified as a cluster of excellence for the application of textile reinforced concrete. The potential hazardousness of the resin used in the impregnation of the fibers commonly applied in textile reinforced concrete was recalled.

The performance of TRC with unresin carbon fibers, concerning control of cracking was assessed through tensile testing. It was found that control of cracking is possible, using unresin carbon fibers, if the load bearing capacity of embedded reinforcement is not less than the cracking load. The tensile behavior of the tested composites was similar to other TRC found in the literature.

A novel system to transfer the load from the testing machine to the specimens was introduced and has proven effective.

The economic and environmental analysis demonstrated a similar performance of TRC with prepreg and with unresin fibers, and both clearly outperformed conventional reinforced concrete. However, TRC with unresin fibers is considered preferable, due to the benefits concerning health, ease of recycling and resistance to deterioration under high temperatures.

The subpar performance of A50 must have been due to less fiber involvement by the matrix. This will be further investigated through a dedicated experimental program encompassing matrices with different workability and cross-section observation using microscopy. Anyway, unlike in conventional reinforced concrete, it is recommended that the mechanical performance of each TRC solution (fiber-matrix combination) should be experimentally evaluated. Such assessment must provide the reinforcement bearing capacity when embedded in the matrix, a fundamental parameter for control of cracking, as addressed in the present research, but also for other design criteria, e.g., the bending ultimate limit state.

## Figures and Tables

**Table 1:** Mix proportions (wt./wt.).

Mix	Cement:Fine Sand:Coarse Sand:Water
A35 ^1^	1:0.4:2.1:0.35
A40 ^2^	1:0.4:2.1:0.41
A50	1:0.4:2.1:0.50
A60	1:0.5:2.5:0.60

^1^ 12 mL of superplasticizer per kg of cement; ^2^ 8 mL of superplasticizer per kg of cement.

**Table 2:** Matrix strength (MPa).

Mix	Compressive Strength	Flexural Strength	Tensile Strength
A35	67.3	11.4	5.4
A40	46.8	8.5	4.0
A50	34.4	7.7	2.8
A60	30.1	6.8	2.5

**Table 3:** Results from direct tension test on textile reinforced concrete (TRC).

Matrix	σ_cr_ (MPa)	F_u_ (N)	ε_u_ (^o^/_oo_)	S_rm_ (mm)
A35	6.5	3406	0.17	-
A40	4.1	3128	7.2	14
A50	3.2	3016	6.8	50
A60	3.4	3189	8.0	13

**Table 4:** Strain energy and ductility.

Matrix	U (J)	Ductility (-)
A35	677	1
A40	7765	83
A50	5533	68
A60	7945	50

**Table 5:** Materials relative cost per unit volume.

Material	Relative Cost
Steel	1
Concrete	0.23
Unresin Carbon Fiber	5
Impregnated Carbon Fiber	25

**Table 6:** Cost analysis.

Solution	CRC ^1^		TRC w/UCF ^1^		TRC w/ICF ^1^	
Material	Quantity (m^3^)	Cost	Quantity (m^3^)	Cost	Quantity (m^3^)	Cost
Steel	35 × 10^−5^	35 × 10^−5^				
Concrete	0.060	1.38 × 10^−2^	0.018	4.14 × 10^−3^	0.018	4.14 × 10^−3^
Carbon Fiber			32.94 × 10^−6^	16.47 × 10^−5^		
Impregnated Carbon Fiber					18.38 × 10^−6^	45.95 × 10^−5^
Total Cost		14.15 × 10^−3^		4.30 × 10^−3^		4.60 × 10^−3^

^1^ CRC: conventional reinforced concrete; UCF: unresin carbon fibers; ICF: impregnated carbon fibers.

**Table 7:** CO_2_ emission ratios (wt./wt.).

Material	CO_2_ Emission Ratio
Steel	1.91
Concrete	0.1
Carbon Fiber	28.7
Impregnation Resin	5.7

**Table 8:** Comparative analysis of CO_2_ emissions (kg).

Solution	CRC ^1^		TRC w/UCF ^1^		TRC w/ICF ^1^	
Material	Quantity (kg)	CO_2_ Emission	Quantity (kg)	CO_2_ Emission	Quantity (kg)	CO_2_ Emission
Steel	2.75	5.25				
Concrete	139.2	13.92	41.8	4.18	41.8	4.18
Carbon Fiber			0.060	1.72	0.033	0.95
Impregnation Resin					0.027	0.15
**Total CO_2_ emission**		19.17		5.90		5.28

^1^ CRC: conventional reinforced concrete; UCF: unresin carbon fibers; ICF: impregnated carbon fibers.
